# Identification of Zoonotic *Balantioides coli* in Pigs by Polymerase Chain Reaction-Restriction Fragment Length Polymorphism (PCR-RFLP) and Its Distribution in Korea

**DOI:** 10.3390/ani11092659

**Published:** 2021-09-10

**Authors:** Jae-Won Byun, Jung-Hyun Park, Bo-Youn Moon, Kichan Lee, Wan-Kyu Lee, Dongmi Kwak, Seung-Hun Lee

**Affiliations:** 1 Animal and Plant Quarantine Agency, Gimcheon 39660, Korea; jaewon8911@korea.kr (J.-W.B.); qiamby@korea.kr (B.-Y.M.); noanoa33@korea.kr (K.L.); 2 College of Veterinary Medicine, Chungbuk National University, Cheongju 28644, Korea; david0132@naver.com (J.-H.P.); wklee@cbu.ac.kr (W.-K.L.); 3 College of Veterinary Medicine, Kyungpook National University, Daegu 41566, Korea; dmkwak@knu.ac.kr

**Keywords:** *Balantioides coli*, *Balantidium coli*, diagnosis, zoonosis, pig, protozoa, PCR-RFLP

## Abstract

**Simple Summary:**

*Balantioides coli* is a protozoan parasite that can infect humans, and its main reservoir is pigs. Recent studies suggested that one of *B. coli* variants, named variant A, has zoonotic potential. Previous studies have reported *B. coli* infection in pigs in different countries; however, the prevalence of the zoonotic variant is limited due to a lack of molecular information. In this study, we developed a molecular technique-based method that could differentiate *B. coli* variant A from B without sequence analysis. Using the method, 174/188 (94.6%) pig fecal samples collected in domestic pigs in Korea were positive for *B. coli*, and of the samples, 62 (33.7%) were the zoonotic variant. To our knowledge, this is the first study to develop a method to differentiate *B. coli* variants A and B without sequence analysis and to assess the molecular epidemiology of *B. coli* in pigs.

**Abstract:**

*Balantioides coli* is a zoonotic protozoan parasite whose main reservoir is pigs. Recent studies have shown that *B. coli* variant A but not B has zoonotic potential. While *B. coli* infection has been reported in different animals and countries, the prevalence of the zoonotic variant is limited due to a lack of molecular information. Therefore, this study investigated the prevalence of *B. coli* in domestic pigs in Korea and assessed its zoonotic potential. A total of 188 pig fecal samples were collected from slaughterhouses in Korea. *B. coli* was identified by microscopy and molecular methods. *B. coli* was identified in 79 (42.9%) and 174 (94.6%) samples by microscopy and polymerase chain reaction (PCR), respectively. This study also developed a PCR-restriction fragment length polymorphism (PCR-RFLP) method to differentiate *B. coli* variant A from B without sequence analysis. Using this method, 62 (33.7%) and 160 (87.0%) samples were positive for variants A and B, respectively, and 48 (26.1%) samples were co-infected with both variants. Sequence and phylogenetic analyses showed a high genetic diversity of *B. coli* in pigs in Korea. To our knowledge, this is the first study to develop a method to differentiate *B. coli* variants A and B without sequence analysis and to assess the molecular epidemiology of *B. coli* in pigs. Continuous monitoring of zoonotic *B. coli* in pigs should be performed as pigs are the main source of human balantidiasis.

## 1. Introduction

*Balantioides coli*, the only ciliate protozoan parasite, can infect humans. The main reservoir of *B. coli* is pigs; however, it also infects other animals including non-human primates, cattle, buffalo, sheep, goats, rodents, and birds [1,2,3,4,5]. With its broad host range, *B. coli* is distributed worldwide, especially in tropical and subtropical areas. *B. coli* was first named *Paramecium coli* in 1857 (reviewed by [2]) and was transferred to the genus *Balantidium* in 1863 (reviewed by [2]). Recent advances in molecular techniques have revealed genetic differences between *B. coli* and other *Balantidium* spp., and *B. coli* has been moved to the genus *Neobalantidium* [4]. As *Neobalantidium* is synonymous with *Balantiodies* as proposed by Alexeieff in 1931, *Balantioides* was used as the correct genus name [6].

The transmission of *B. coli* to its host occurs via the fecal–oral route, and *B. coli* parasitizes the large intestine, cecum, and colon of its hosts [2,7]. Humans and animals are infected by ingesting *B. coli* cysts directly or indirectly through contaminated food and water. Infection does not generally cause clinical symptoms in immunocompetent animals or humans. However, in immunocompromised hosts such as patients with AIDS or co-infection with other pathogens, *B. coli* causes diarrhea, malnutrition, and other gastrointestinal symptoms [2,8]. While few clinical cases have been reported in animals, cases of human balantidiasis have been reported with dysentery as the main symptom [2,5].

There is no standardized diagnostic method for *B. coli* and general coproscopic examination methods based on floatation or sedimentation are used [7]. Due to its distinctive size and morphological characteristics, the diagnosis of *B. coli* is straightforward. However, microscope-based diagnosis has disadvantages such as low sensitivity, inability to evaluate genetic characteristics, and difficulty in differentiating morphologically similar pathogens (e.g., *Buxtonella* spp.) [2,8]. 

Recent advancements in molecular techniques have allowed for the investigation of different molecular characteristics of *B. coli*. Ponce-Gordo et al. [1] performed molecular analysis of *B. coli* based on the 18S-rRNA–ITS1–5.8S-rRNA–ITS2 regions (hereafter, ITS region) and reported at least two genetic variants; namely, variants A and B. Of these, *B. coli* variant A is considered to be zoonotic; however, studies on its association with animal species and distribution are insufficient due to a lack of molecular information [9]. Previous PCR-based studies have identified genetic variants of *B. coli* using sequence analysis with cloning, which requires significant labor, time, and cost [1,8]. To evaluate zoonotic *B. coli* in many samples from different regions, more precise, labor-, time-, and cost-efficient methods need to be developed. PCR-restriction fragment length polymorphism (PCR-RFLP) is one of the most commonly used tools to analyze the molecular characteristics of pathogens. It has been used for various purposes including species differentiation, pathogenicity prediction, and genotypic analysis [10,11,12].

Previous studies have reported the presence of *B. coli* infection in humans and pigs in Korea, some of which were related to clinical cases [13,14,15,16]. However, the studies diagnosed *B. coli* infection based on microscopic examination without molecular analysis. Therefore, molecular information on *B. coli* in domestic pigs and humans in Korea is lacking and the zoonotic potential of *B. coli* in Korea is unknown. Considering reports of human balantidiasis and the presence of *B. coli* in animals in Korea, the distribution and prevalence of zoonotic *B. coli* requires evaluation.

Therefore, the purpose of this study was two-fold. First, we developed a PCR-RFLP method to differentiate *B. coli* variants A and B. Second, we investigated the prevalence of *B. coli* and its genetic variants in pigs in Korea and molecularly characterized them.

## 2. Materials and Methods

### 2.1. Collection of Pig Fecal Samples

**Figure 1:**
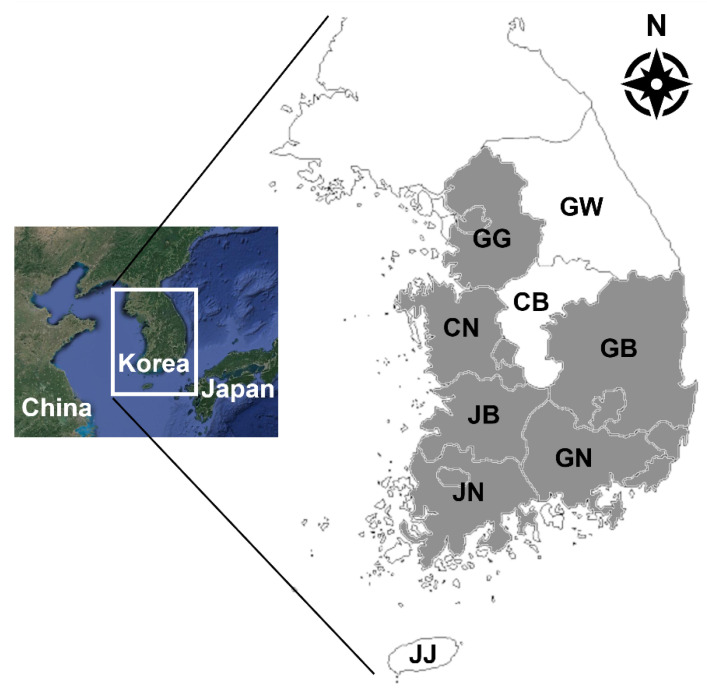
Map of Korea. The sites of pigs reared are indicated in gray. GG, Gyeonggi-do; GW, Gangwon-do; CN, Chungcheongnam-do; CB, Chungcheongbuk-do; GB, Gyeongsangbuk-do; GN, Gyeongsangnam-do; JB, Jeollabuk-do; JN, Jeollanam-do; JJ, Jeju-do.

From May to November 2020, 188 pig fecal samples from 32 farms were collected from slaughterhouses in Korea (Figure 1). Information on the rearing regions and sample collection dates were recorded. All pigs were raised for meat production and were approximately six months of age. Fresh fecal samples were collected directly from the large intestine by dissection to avoid cross- or environmental contamination, transported to the College of Veterinary Medicine, Chungbuk National University, Korea, and stored at 4 °C.

### 2.2. Microscopical Identification of B. coli

The transported fecal samples were homogenized using a sterilized wooden stick, and 1 g and 200 mg of fecal samples were taken for microscopic examination and DNA extraction, respectively. Microscopic examination was performed using the fecal flotation technique with sodium nitrate, as previously described [17]. 

### 2.3. DNA Extraction, PCR, Cloning, and Sequencing

Genomic DNA was extracted from the fecal samples using a QIAamp Fast DNA Stool Mini Kit (Qiagen, Hilden, Germany) according to the manufacturer’s instructions. The extracted DNA was kept at –20 °C until further analysis. 

For molecular diagnosis, PCR targeting the ITS region of *B. coli* was performed as previously described [1]. In brief, PCR was performed using AccuPower ^®^ HotStart PCR PreMix (Bioneer, Korea) containing 2 μL template DNA, 1 μL of each forward and reverse primer (0.5 μM final concentration), and distilled water to a final volume of 20 μL. The PCR conditions were as follows: initial denaturation for 5 min at 94 °C; 30 cycles of 1 min at 94 °C, 1 min at 56 °C, and 1 min at 72 °C; and final extension for 5 min at 72 °C. After verification of the PCR conditions using a positive control, PCR was performed without a positive control to avoid cross-contamination, with distilled water included as a negative control in each experiment. The expected amplicon sizes were 528- or 537-bp according to the *B. coli* variant. The PCR products were visualized by gel electrophoresis.

For sequencing and phylogenetic analysis, 11 samples were randomly selected and amplicons of the expected size were cut, purified, and cloned into *E. coli* DH5α using the pGEM-T easy vector system (Promega, Madison, WI, USA). At least three colonies per sample were selected and sequenced using a universal primer set (M13F and M13R) by Macrogen (Daejeon, Korea). The sequences obtained were aligned using MEGA 7.0. and the species were determined using a BLASTn search [18,19].

### 2.4. PCR-RFLP

To differentiate *B. coli* variant A from variant B, a PCR-RFLP technique was developed using the restriction enzymes *Apo*I and *PflM*I. The reference sequences of *B. coli* variant A (JQ073359) and variant B (JQ073358) were obtained from the GenBank database. In silico PCR-RFLP predicted that *Apo*I cut *B. coli* variant A into 227-, 135-, 70-, 70-, and 35-bp fragments and variant B into 293- and 235-bp fragments. *PflM*I had no cut site in *B. coli* variant A and 312- and 216-bp fragments in *B. coli* variant B (Table 1).

The PCR-RFLP reaction was performed as follows: 10 μL PCR products, five units restriction enzyme (New England Biolabs, Ipswich, MA, USA), 2 μL 10X buffer (New England Biolabs), and distilled water up to a total volume of 20 μL. The mixtures containing *Apo*I and *PflM*I were incubated at 50 °C and 37 °C, respectively, for one hour. 

### 2.5. Sequence and Phylogenetic Analyses

For molecular characterization, sequence analysis was performed using DnaSP v6, while phylogenetic analysis was performed using MEGA 7.0 [20]. The phylogenetic tree was constructed using the maximum likelihood method, with verification of the bootstrap values by 500 bootstrap replications. The analysis also included sequences reported from other animal species and countries obtained from the GenBank database. *Spathidium amphoriforme* (AF223570) was included as an outgroup.

## 3. Results

### 3.1. Prevalence of B. coli by Microscopy and PCR

Microscopy identified *B. coli* in 79 (42.9%) out of 184 fecal samples. All identified *B. coli* were in the cyst stage and not the trophozoite stage (Figure S1). Through PCR, 174 (94.6%) out of 184 samples were positive for *B. coli*.

All the sampled regions showed a high prevalence, ranging from 75.0% to 100% (Table 2). In addition, all farms had at least one *B. coli-*infected pig (data not shown).

### 3.2. PCR-RFLP and Prevalence According to Variant

**Figure 2:**
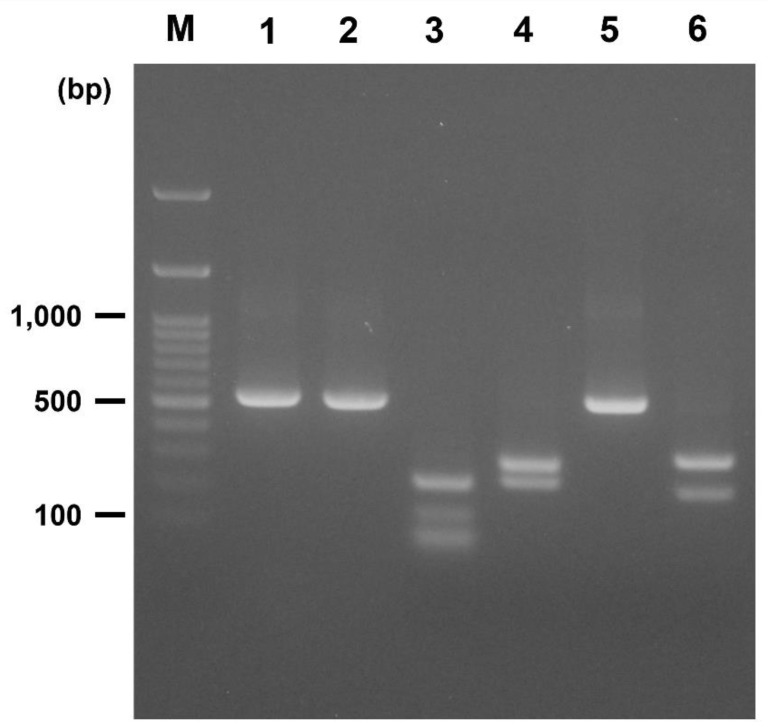
Polymerase chain reaction-restriction fragment length polymorphism (PCR-RFLP) results of *Balantioides coli* variants A and B with restriction enzyme digests by *Apo*I and *PflM*I. M, marker; 1, *B. coli* variant A (537-bp); 2, *B. coli* variant B (528-bp); 3, *B. coli* variant A treated with *Apo*I (227, 135, 70, 35-bp); 4, *B. coli* variant B treated with *Apo*I (293, 235-bp); 5, *B. coli* variant A treated with *PflM*I (537-bp); 6, *B. coli* variant B treated with *PflM*I (312, 216-bp). Note that the 35-bp fragment is not visible.

PCR-RFLP using *Apo*I and *PflM*I showed clear differentiation between *B. coli* variants A and B (Figure 2). Using PCR-RFLP, 62 (33.7%) and 160 (87.0%) samples were positive for variants A and B, respectively, and 48 (26.1%) samples were co-infected with both variants (Table 3). 

### 3.3. Sequence and Phylogenetic Analyses

**Figure 3:**
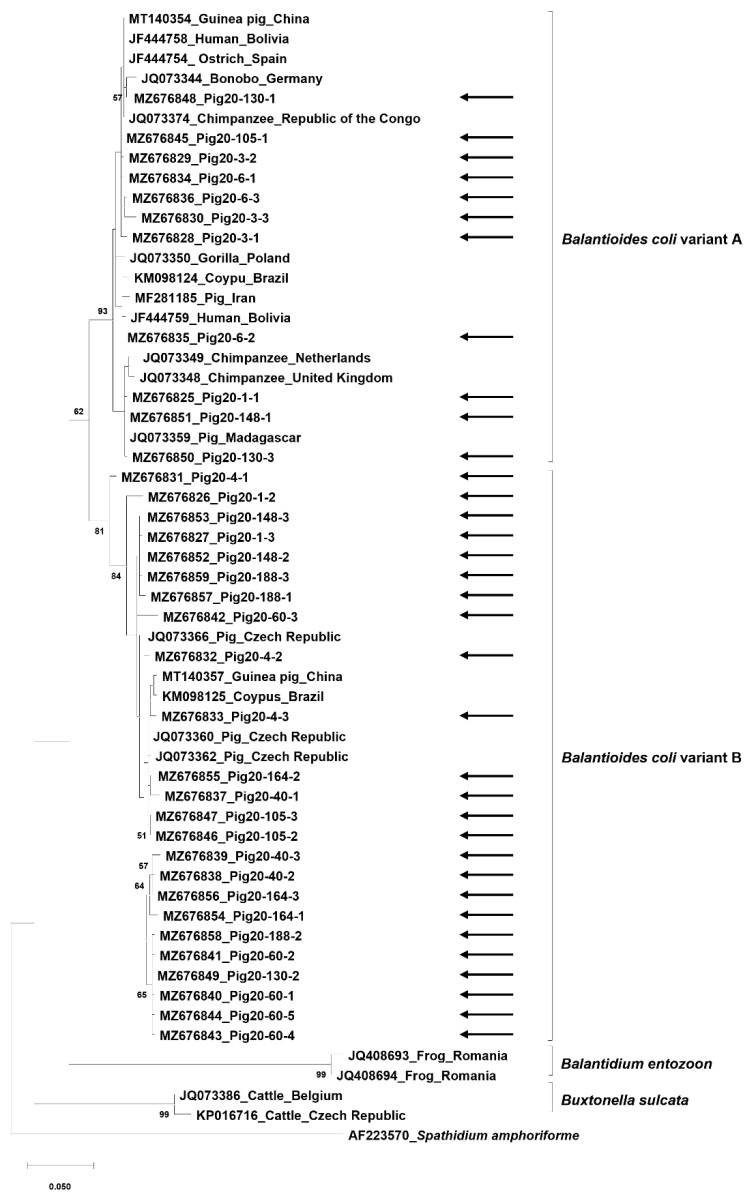
Phylogenetic analysis of *Balantioides coli* in pigs in Korea. The tree was constructed using MEGA 7.0 and the maximum-likelihood method with 500 replications. The sequences analyzed in this tree were obtained from the GenBank database and are described with their accession numbers, hosts, and isolated countries. The sequences identified in this study are indicated with arrows. *Spathidium amphoriforme* (AF223570) is included as an outgroup. Bootstrap values less than 40 were omitted.

Of the 11 positive samples, 35 colonies were selected and sequences were successfully obtained. Sequence analysis showed 92.9–100% intraspecies identity. The sequences showed 63 polymorphic sites including in 19 and 42 positions in variants A and B, respectively. In addition, 33 haplotypes were identified, with 11 and 23 haplotypes in variants A and B, respectively. Phylogenetic analysis showed that both *B. coli* variants were present in pigs in Korea (Figure 3). All sequences obtained in this study were submitted to the GenBank database (Accession Nos. MZ676825-MZ676859).

There was agreement between the results obtained by PCR-RFLP and sequencing. Not all variants were identified in some samples co-infected with *B. coli* variants; however, in cases of samples infected by a single *B. coli* variant, all the clones contained the corresponding variant only.

## 4. Discussion

*B. coli* is distributed worldwide, and its main host is pigs [7]. Previous studies have reported the prevalence of *B. coli* in domestic pigs in different countries including 46.4% and 84.1% in Japan (155/334 and 212/252, respectively), 93.0% in India (93/100), 16.8% in China (94/560), 40.0% in Bangladesh (44/110), 1.6%, in Turkey (4/238), 51.5% in Nigeria (207/402), 64.1% in Kenya (196/306), 0.7% in Germany (2/287), 61.6% in Italy (149/242), and 60.9% in Brazil (236/387) [8,21,22,23,24,25]. The prevalence in domestic pigs is highly variable, ranging from 0.7% to 93.0%, and might be affected by the breeding system, hygiene conditions, season, diagnostic method, and climate. Even in microscopic examinations, performance can vary according to techniques, materials, and the investigator’s experience [7,26]. As shown in this study, molecular methods generally show higher sensitivity than microscopic examinations. Previous studies were mainly based on the microscopic method; therefore, the true prevalence in those studies may have been underestimated. 

Few studies have evaluated *B. coli* in pigs in Korea. To our knowledge, only two studies have investigated the prevalence of *B. coli* in pigs by microscopy, with reported prevalences of 79.4% (108/136) and 66.6% (263/395) from samples collected in Chungcheongnam-do and Chungju city, respectively [13,27]. However, due to the lack of molecular information, the zoonotic potential of *B. coli* has not been evaluated.

Even before applying molecular techniques to *B. coli*, the role of pigs in the transmission of *B. coli* to humans is well known [28]. In developing countries, human balantidiasis is mainly caused by poor sanitation systems and ingestion of *B. coli*-contaminated food and water [28,29,30]. Molecular techniques make it possible to evaluate the role of animals and the zoonotic potential of *B. coli* according to their molecular characteristics. Ponce-Gordo et al. first suggested the zoonotic potential of *B. coli* variant A, which was identified in Bolivian patients and pigs [9]. Subsequent studies investigated the presence of *B. coli* variant A in animals from other countries. To date, *B. coli* variant A has been identified only in humans, non-human primates (gorillas and chimpanzees), guinea pigs, ostriches, and pigs [3,4,8,9]. Of these, *B. coli* from guinea pig was identical to the human-genotype, suggesting the importance of animals in the transmission of human balantidiasis [3].

Previous studies identified *B. coli* variant A based on sequence analysis after cloning; however, it is not an appropriate method to evaluate the true prevalence of each *B. coli* variant. As Ponce revealed, both variants A and B can exist in a single *B. coli* cell, and the results obtained by cloning are affected by probability [9]. To overcome this limitation, this study developed PCR-RFLP to evaluate the true prevalence of both *B. coli* variants in pigs. 

This study is the first to show the molecular prevalence of *B. coli* variants A and B in pigs in Korea, with a predominance of variant B. This result is consistent with those of previous studies in China, which reported a higher prevalence of *B. coli* variant B compared to variant A [8,31]. To date, few studies have investigated *B. coli* based on molecular techniques, and the distribution of zoonotic *B. coli* worldwide is uncertain. More studies should be conducted with molecular information on *B. coli* variants in different countries.

Cases of human balantidiasis have been reported sporadically in Korea, with clinical signs varying from asymptomatic to dysentery [14,16,32,33]. Human balantidiasis has also been reported in other countries including Europe, Canada, and the U.S. [34,35,36,37]. The main clinical signs of balantidiasis are gastrointestinal disorders including diarrhea and abdominal pain [2]; however, infection and clinical signs are not confined to the gastrointestinal system. Previous studies have reported *B. coli* infection in different organs including the lungs, liver, genitourinary tract, cervical cord, brain, ascitic fluid, and eyes [2,14,16,32,33]. Unfortunately, these studies were mainly based on microscopic examination, histopathologic diagnosis; thus, the genetic variants causing human balantidiasis have not been identified. A recent study reported a case of human balantidiasis with dysentery among workers on pig farms in China [38]. Although the study did not also confirm the genetic variant, the findings demonstrated the importance of pigs as a source of *B. coli* infection in humans. 

Different risk factors have been suggested for *B. coli* infection including both host and environmental factors [2,7]. Sex and age are the most common host-related risk factors associated with the disease. To our knowledge, there is no consensus on risk factors for *B. coli* infection. For example, different studies have reported different prevalence according to age group, some of which were statistically significant, while others were not [8,24,39,40,41]. In addition, the results of risk factor analysis for sex are contradictory [24,40,41]. Well-designed and controlled studies are required for risk factor analysis.

*B. coli* is generally considered a non-pathogenic or opportunistic pathogen in animals and humans because of its high prevalence and low clinical cases. Comorbidities such as bacterial and viral infections may be related to the onset of clinical symptoms [2]. A recent study showed that *B. coli* infection alters gut microbiota by increasing *Escherichia-Shigella* and *Campylobacter* and decreasing *Ruminococcaceae* and *Clostridiaceae* [42], and the authors stated that *B. coli* needs to be considered as a pathogenic or opportunistic pathogenic parasite [42]. 

The results of the sequence and phylogenetic analyses in this study demonstrated the high level of genetic diversity of *B. coli* in pigs in Korea. Variants A and B are currently considered the main classification criteria in *B. coli*; however, Ponce-Gordo et al. classified the variants in more detail including A0, A1, A2, B0, and B1 [9]. Previous studies have unsuccessfully attempted to identify an association between genetic variants and animal species [3,4,9]; however, the accumulation of molecular information from different animals using different genetic markers may be helpful. 

## 5. Conclusions

To our knowledge, this is the first study to develop a method to identify the genetic variants of *B. coli* in pigs without sequence analysis and to investigate the molecular epidemiology of *B. coli* in pigs. The results of this study showed the high prevalence of *B. coli* in pigs in Korea and the predominance of genetic variant B. Moreover, *B. coli* variant A, which has zoonotic potential, is widely distributed in Korea. Although the pathogenicity of *B. coli* in pigs is not critical, pigs are the main source of human balantidiasis. Therefore, molecular-based investigation of zoonotic *B. coli* in pigs in different countries is required.

## Figures and Tables

**Table 1:** Polymerase chain reaction-restriction fragment length polymorphism (PCR-RFLP) results of *Balantioides coli* variants A and B digested with restriction enzymes *Apo*I and *PflM*I.

*B. coli*	PCR Product (bp)	Restriction Enzyme (bp)	
		*Apo*I	*PflM*I
Variant A	537	227, 135, 70, 70, 35	537
Variant B	528	293, 235	312, 216

**Table 2:** Prevalence of zoonotic *Balantioides coli* in pigs in Korea according to province.

Province	No. of Samples	No. of Positive (%)		
		Microscopy	PCR	*B. coli* Variant A
Gyeonggi-do	47	23 (48.9)	41 (87.2)	14 (34.1)
Chungcheongnam-do	8	5 (62.5)	6 (75.0)	0 (0.0)
Gyeongsangbuk-do	45	14 (31.1)	45 (100)	24 (53.3)
Gyeongsangnam-do	60	21 (35.0)	58 (96.7)	14 (24.1)
Jeollabuk-do	16	11 (68.8)	16 (100)	8 (50.0)
Jeollanam-do	8	5 (62.5)	8 (100)	2 (25.0)
Total	184	79 (42.9)	174 (94.6)	62 (33.7)

**Table 3:** Proportions of *Balantioides coli* genetic variants in pigs in Korea.

*B. coli* Infection	No. of Samples (%)
Co-infection (variant A + variant B)	48 (26.1)
Single infection (variant A)	14 (7.6)
Single infection (variant B)	112 (60.9)
Non	10 (5.4)
Total	184

## Data Availability

The data presented in this study are contained within this article and all sequences obtained in this study were submitted to the GenBank Database (Accession Nos. MZ676825-MZ676859).

## Supplementary Materials

The following are available online at https://www.mdpi.com/article/10.3390/ani11092659/s1, Figure S1: Microscopic identification of Balantioides coli in pig feces. (A) Without staining, showing a bean-shaped macronucleus (arrow). (B) Lugol’s iodine staining showing the cyst wall (arrowhead). Bar = 50 μm.
