# Supplementary material for: Identification of Zoonotic Balantioides coli in Pigs by Polymerase Chain Reaction-Restriction Fragment Length Polymorphism (PCR-RFLP) and Its Distribution in Korea
Source: Animals (Basel). 2021 Sep 10;11(9):2659. doi: 10.3390/ani11092659 (PMC8465230; doi:10.3390/ani11092659)

Supplementary Materials:

Figure S1: Microscopic identification of *Balantioides coli* in pig feces. (**A**) Without staining, showing a bean-shaped macronucleus (arrow). (**B**) Lugol's iodine staining showing the cyst wall (arrowhead). Bar = 50 µm.

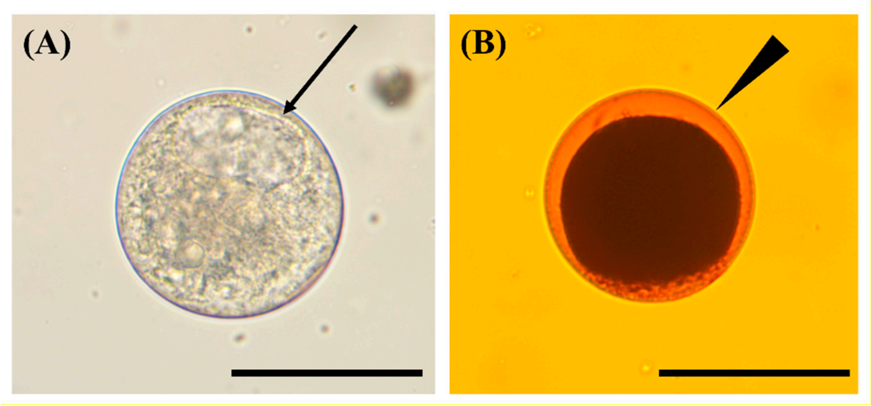

Supplement: Supplementary file 1 [file animals-11-02659-s001.zip › animals-1355212-supplementary.pdf]
